# Long-Read Sequencing Reveals Rapid Evolution of Immunity- and Cancer-Related Genes in Bats

**DOI:** 10.1093/gbe/evad148

**Published:** 2023-09-20

**Authors:** Armin Scheben, Olivia Mendivil Ramos, Melissa Kramer, Sara Goodwin, Sara Oppenheim, Daniel J Becker, Michael C Schatz, Nancy B Simmons, Adam Siepel, W Richard McCombie

**Affiliations:** Simons Center for Quantitative Biology, Cold Spring Harbor Laboratory, Cold Spring Harbor, New York, USA; Cold Spring Harbor Laboratory, Cold Spring Harbor, New York, USA; Cold Spring Harbor Laboratory, Cold Spring Harbor, New York, USA; Cold Spring Harbor Laboratory, Cold Spring Harbor, New York, USA; American Museum of Natural History, Institute for Comparative Genomics, New York, New York, USA; School of Biological Sciences, University of Oklahoma, Norman, Oklahoma, USA; Simons Center for Quantitative Biology, Cold Spring Harbor Laboratory, Cold Spring Harbor, New York, USA; Departments of Computer Science and Biology, Johns Hopkins University, Baltimore, Maryland, USA; Department of Mammalogy, Division of Vertebrate Zoology, American Museum of Natural History, New York, New York, USA; Simons Center for Quantitative Biology, Cold Spring Harbor Laboratory, Cold Spring Harbor, New York, USA; Cold Spring Harbor Laboratory, Cold Spring Harbor, New York, USA

**Keywords:** cancer resistance, Chiroptera, comparative genomics, immunity, long reads, viral tolerance

## Abstract

Bats are exceptional among mammals for their powered flight, extended lifespans, and robust immune systems and therefore have been of particular interest in comparative genomics. Using the Oxford Nanopore Technologies long-read platform, we sequenced the genomes of two bat species with key phylogenetic positions, the Jamaican fruit bat (*Artibeus jamaicensis*) and the Mesoamerican mustached bat (*Pteronotus mesoamericanus*), and carried out a comprehensive comparative genomic analysis with a diverse collection of bats and other mammals. The high-quality, long-read genome assemblies revealed a contraction of interferon (IFN)-α at the immunity-related type I IFN locus in bats, resulting in a shift in relative IFN-ω and IFN-α copy numbers. Contradicting previous hypotheses of constitutive expression of IFN-α being a feature of the bat immune system, three bat species lost all IFN-α genes. This shift to IFN-ω could contribute to the increased viral tolerance that has made bats a common reservoir for viruses that can be transmitted to humans. Antiviral genes stimulated by type I IFNs also showed evidence of rapid evolution, including a lineage-specific duplication of IFN-induced transmembrane genes and positive selection in *IFIT2*. In addition, 33 tumor suppressors and 6 DNA-repair genes showed signs of positive selection, perhaps contributing to increased longevity and reduced cancer rates in bats. The robust immune systems of bats rely on both bat-wide and lineage-specific evolution in the immune gene repertoire, suggesting diverse immune strategies. Our study provides new genomic resources for bats and sheds new light on the extraordinary molecular evolution in this critically important group of mammals.

**Significance**

Bats are known for their robust immune systems and cancer resistance, but comparative genomics studies of these unique adaptations have been limited by low sample sizes and incomplete short-read genomes. The analysis of 15 bat genomes, including 8 contiguous long-read genomes, showed a shift in antiviral interferon (IFN)-α and IFN-ω gene copy numbers in bats, as well as positive selection in antiviral genes, tumor suppressors, and DNA-repair genes.

## Introduction

Bats (order Chiroptera) form the second largest order of mammals and are known for a wide variety of remarkable adaptations including powered flight (Simmons et al. 2008), laryngeal echolocation (Simmons and Geisler 1998; Moss and Surlykke 2001), unusual longevity (Wilkinson and Adams 2019), and low rates of cancer (Wang et al. 2011). Bats are also hosts of diverse viruses (Calisher et al. 2006; Olival et al. 2017) and have played roles in outbreaks of emerging zoonotic viruses including Marburg virus (Amman et al. 2012), Nipah virus (Pulliam et al. 2012), and severe acute respiratory syndrome coronavirus 1 (SARS-CoV-1) (Li et al. 2005), either through direct human contact or via bridge hosts. Bats may also have played a role in the emergence of SARS-CoV-2, as closely related progenitor viruses have been detected in wild bats (Andersen et al. 2020; Holmes et al. 2021; Pekar et al. 2022). The ability of bats to tolerate viral infections may stem from unusual features of their innate immune response (Pavlovich et al. 2018). Together, these adaptations make bats a powerful system for investigating a wide variety of genotype-to-phenotype relationships, including several with implications for human health. For example, by better understanding the mechanisms of the bat immune system that allow them to tolerate viral infections (Wang et al. 2011), researchers may be better able to prevent zoonotic outbreaks (Wang et al. 2021; Becker et al. 2022). In addition, comparative genomic analyses of bats and cancer-susceptible mammals may shed new light on the causes of cancer and links between cancer and immunity (Gonzalez et al. 2018). Importantly, such studies of bats and other nonmodel organisms are highly complementary to studies based on mouse models, which are far more amenable to experimental manipulation but exhibit fewer natural adaptations relevant to human disease.

With these goals in mind, investigators have sequenced and assembled the genomes of at least 44 bat species over the past decade (supplementary table S1, Supplementary Material online). Recently, sequencing efforts in bats have been accelerated by the Bat1K global genome consortium (Teeling et al. 2018), DNA Zoo (Dudchenko et al. 2017), and Vertebrate Genome Project (Rhie et al. 2021). These new genome sequences have revealed numerous intriguing features of the immune systems of bats (Zhang et al. 2013; Escalera-Zamudio et al. 2015; Pavlovich et al. 2018; Zepeda Mendoza et al. 2018; Hawkins et al. 2019; Gorbunova et al. 2020; Jebb et al. 2020; Irving et al. 2021; Moreno Santillán et al. 2021). In particular, several genes with key roles in the innate immune system appear to have adaptively evolved in bats, including primary lines of inducible host defenses such as pathogen sensors (Escalera-Zamudio et al. 2015; Jiang et al. 2017), type I interferons (IFNs) (Zhou et al. 2016; Pavlovich et al. 2018), and antiviral genes (Fuchs et al. 2017). Specifically, bats have lost the mammalian PYHIN DNA-sensing gene family (Zhang et al. 2013; Ahn et al. 2016), they show evidence of positive selection in pathogen-sensing Toll-like receptors (TLRs) (Jiang et al. 2017), and they display copy number variation in type I IFN cytokines (Zhou et al. 2016; Pavlovich et al. 2018), which are induced by TLRs. Bat-specific modifications in immune response, tumor suppressors, DNA damage checkpoint-DNA repair pathway genes (Zhang et al. 2013), and growth hormone (Seim et al. 2013) may be associated with cancer resistance. It is thought that these adaptations in innate immunity and cancer resistance may have arisen as a result of coevolution of bats with viruses (Taylor et al. 2011; Jebb et al. 2020) and that a need for enhanced DNA repair in the face of elevated reactive oxygen species (ROS) may have been a consequence of powered flight (Zhang et al. 2013).

In this study, we augment previously existing genome sequences with new Oxford Nanopore Technologies (ONT)-based long-read assemblies for the Jamaican fruit bat (*Artibeus jamaicensis*) and the Mesoamerican mustached bat (*Pteronotus mesoamericanus*) (supplementary fig. S1, Supplementary Material online). Both species belong to Noctilionoidea, a Neotropical superfamily that comprises ∼16% of global bat diversity (www.batnames.org). *Artibeus jamaicensis* is one of the most common Neotropical mammals and a model species in mammalian research (Larsen et al. 2007) including work on bat immunology (Cabrera-Romo et al. 2014; Munster et al. 2016; David et al. 2022). It is a member of the family Phyllostomidae, arguably the most ecologically diverse lineage of living mammals (Fleming et al. 2020). *Pteronotus mesoamericanus* is a representative of the family Mormoopidae, the less well-studied sister group of the species-rich phyllostomids to which *A*. *jamaicensis* belongs (Rojas et al. 2016), making *P*. *mesoamericanus* the first long-read sequenced species in a critical outgroup for study of evolutionary changes in the phyllostomid radiation. Here, we present a comprehensive analysis of these genome sequences together with 13 previously assembled bats and other mammalian genomes. We aim to leverage the high-quality, long-read assemblies to enable accurate and complete characterization of gene duplications and losses and of genomic repeats (Vollger et al. 2019; Halo et al. 2021; Rhie et al. 2021; Blumer et al. 2022). The benefits of long-read assemblies are of particular value in studies of mammalian immunity-related genes (O’Connor and Cornwallis 2022), many of which fall in highly repetitive genomic regions including large arrays of duplicated genes (He et al. 2021). Our comparative genomic analysis of these genome sequences, which have been released as a public resource, provides several new insights into unique features of innate immune response and cancer resistance in bats.

## Results

### Genomic Structure of *A*. *jamaicensis* and *P*. *mesoamericanus*

Recent work in bats has generated several long-read genome assemblies (Pavlovich et al. 2018; Jebb et al. 2020; Moreno Santillán et al. 2021; Blumer et al. 2022), better enabling the study of complex regions. In this case, we were able to leverage the ONT long-read sequencing platform and an optimized flye-PEPPER-POLCA (Kolmogorov et al. 2019; Zimin and Salzberg 2020; Shafin et al. 2021) assembly and polishing strategy (see Materials and Methods) to generate reference-quality assemblies for *A*. *jamaicensis* and *P. mesoamericanus* with contig N50 values of 28–29 Mb (supplementary fig. S2 and supplementary table S2, Supplementary Material online) and POLCA consensus accuracy >99.99%. Using EVidenceModeler, we annotated 21,621 genes in *A. jamaicensis* and 21,269 genes in *P. mesoamericanus*. Based on the Benchmarking Sets of Universal Single-Copy Orthologs (BUSCO) protein assessment of our annotations, the gene sets in both bats are highly complete at 98.3% and 98.2%, respectively, comparable with the values of 97.4–98.3% reported for six recent PacBio-based bat assemblies (supplementary fig. S2, Supplementary Material online). Notably, all of these long-read bat assemblies have BUSCO scores approaching those of the human (99.9%) and mouse (99.9%) genomes. Orthofinder orthology detection produced 19,935 orthogroups for 15 bats and 5 outgroup mammals, of which 12,836 single-copy orthogroups were set aside for our positive selection analysis (below). Total fractions of 39.2% and 37.9% of the *A*. *jamaicensis* and *P. mesoamericanus* genomes consisted of repeats, respectively, with 0.4% in each genome attributed to recently active transposons including hAT, TcMariner, and piggyBac elements (supplementary fig. S2 and supplementary table S3, Supplementary Material online). We also detected nonretroviral endogenous viral elements, predominantly derived from Bornaviridae and Parvoviridae (supplementary table S4, Supplementary Material online). We provide our annotations, aligned evidence, and multiple genome alignments as a public University of California Santa Cruz (UCSC) genome browser instance (http://compgen.cshl.edu/bat).

### Gene Family Expansion and Contraction Analysis

Changes in gene-family size have played an important role in shaping the immune systems of bats (Zhang et al. 2013; Ahn et al. 2016). To facilitate further analysis of gene-family expansions and contractions, we focused on our new ONT-based assemblies and the previously published long-read bat genome sequences. By comparing these bat genomes with mammalian outgroups (human, mouse, dog, pig, and horse), we identified 14 expanded gene families and 105 contracted gene families in the most recent common ancestor of bats (hereafter, the “bat MRCA”; supplementary table S5, Supplementary Material online). Thirty-nine of these 119 gene families changing size in the bat MRCA were related to immune system processes (Fisher's exact test, *P* = 2.1e−4), including the previously identified PYHIN gene family (PTHR12200) (Ahn et al. 2016), which was absent in all bats including the 2 newly sequenced species. There were significant differences in gene birth–death rates (λ) among Yangochiroptera, Yinpterochiroptera, and nonbat mammals (see Materials and Methods). Yangochiroptera showed the highest rate of gene birth–death, with a λ value of 0.0017 per gene per million years compared with the 0.0008 estimated for Yinpterochiroptera and 0.0005 for nonbat mammals.

### The type I IFN locus is contracted in bats through losses of IFN-α but not IFN-ω

The type I IFN immune response is a critical component of the mammalian innate immune system and is responsible for activating the expression of a battery of antiviral genes following induction by pathogen-sensing components such as PYHINs, TLRs, and *cGAS*-*STING* (McNab et al. 2015). Previous comparative analyses of the type I IFN locus have shown that it is highly structurally variable in bats and other mammals (Zhou et al. 2016), with some bats, such as *Pteropus alecto,* showing a contraction (Zhou et al. 2016), whereas others such as *Rousettus aegyptiacus* (Pavlovich et al. 2018), *Pteropus vampyrus*, and *Myotis lucifugus* (Kepler et al. 2010) show evidence of expansions. However, this locus is generally large and highly duplicated across mammals (e.g., in humans, it spans ∼400 kb and contains 16 IFN genes, including 13 IFN-α genes and 1 IFN-ω gene), making it challenging to assemble and analyze.

**Fig. 1.**
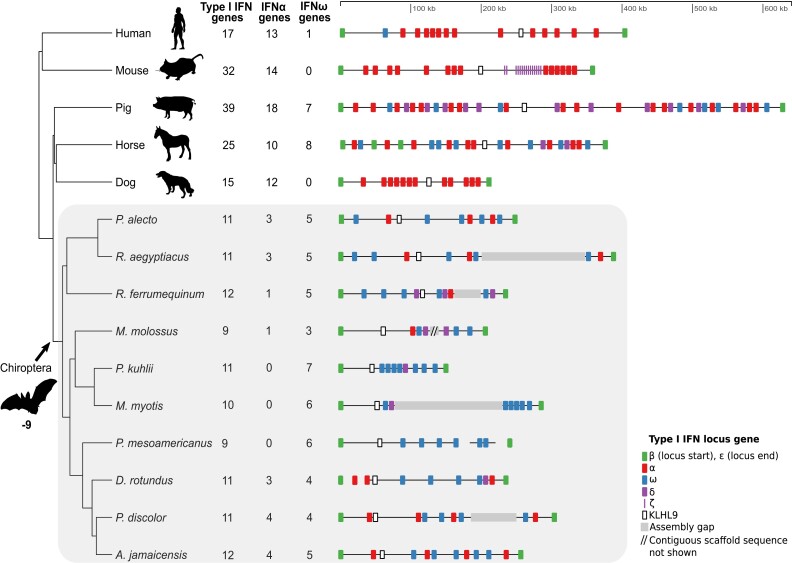
Contraction of the type I IFN locus in bats compared with other mammals. A loss of nine genes in the bat MRCA was estimated by CAFE (Viterbi method, *P* = 2.7e−15). The reduction in locus size occurred together with a significant loss of IFN-α genes but not IFN-ω genes in the bat MRCA (Fisher's exact test, *P* = 4.5e−11, odds ratio = 10.80). The type I IFN loci in bats (gray background) are shown for long-read–based assemblies as well as a BAC-based locus assembly of *P. alecto* (Zhou et al. 2016) and an Illumina-based assembly of *D. rotundus* (Zepeda Mendoza et al. 2018). *M. molossus*, *Molossus molossus*.

Using our expanded set of long read-based bat genomes, we found evidence of a major contraction (−9 genes; Viterbi method (De Bie et al. 2006), *P* = 2.7e−15) of the type I IFN gene family in the bat MRCA (fig. 1; supplementary fig. S3, Supplementary Material online). Comparison of the IFN locus in our assembly with this locus in an earlier short-read assembly of *A*. *jamaicensis* shows that the short-read assembly is both fragmented and incomplete in gene and noncoding sequence content, highlighting the value of long reads for analysis of complex loci (supplementary fig. S4, Supplementary Material online). We found that this contraction was driven specifically by loss of IFN-α genes, with gene counts of 0–4 in bats compared with 10–18 in the outgroup mammals. In contrast, IFN-ω gene counts in the bat ancestor were largely unchanged (Viterbi method, *P* = 0.53), ranging from three to seven in bats and zero to eight in other mammals. As a consequence, IFN-ω was 11-fold enriched relative to IFN-α in bats compared with the other mammals (Fisher's exact test, *P* = 4.5e-11, odds ratio = 10.80), and this enrichment was observed in every bat species (supplementary table S6, Supplementary Material online). Considering the relative ligand-binding and antiproliferative properties of IFN-ω and IFN-α (Jaks et al. 2007; Thomas et al. 2011), these changes in gene number could potentially be responsible for more potent responses to viral infections in bats relative to other mammalian orders (see Discussion).

### Antiviral IFN-Induced Transmembrane Genes Are Expanded in Yangochiroptera Bats

**Fig. 2.**
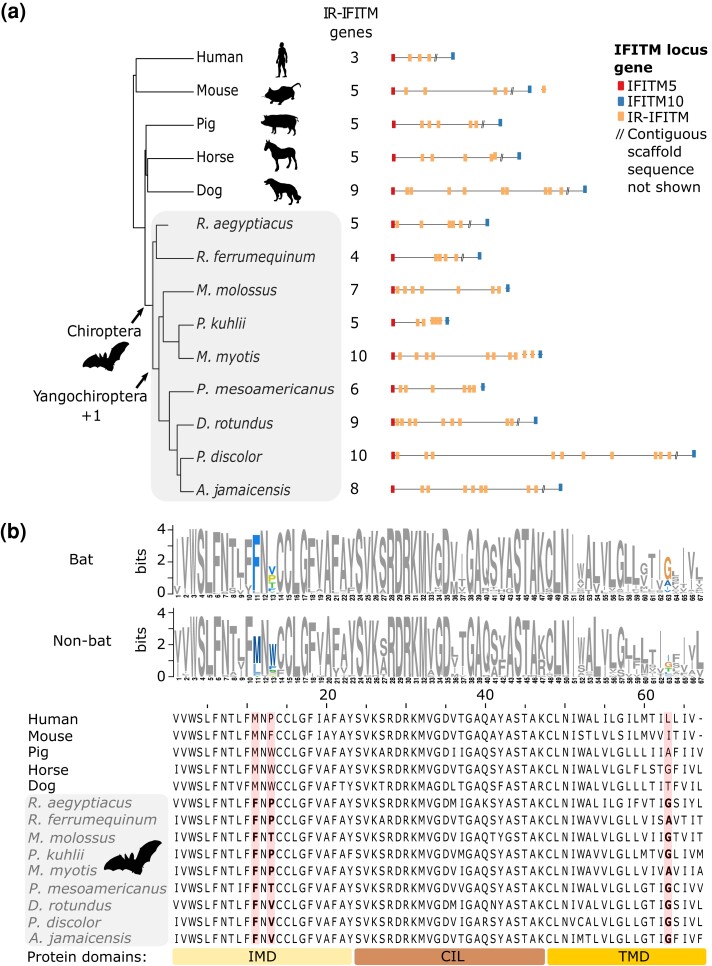
IFITM gene family expansion and positive selection associated with the bat antiviral immune response. (*a*) Phylogeny of bats and other mammals, showing a significant increase in gene copy number at the IFITM locus in Yangochiroptera bats based on CAFE analysis. IR-IFITM genes are shown in yellow. (*b*) Three codon sites in the IFITM transmembrane domains IMD and TMD in bats show evidence of positive selection (see also supplementary table S7, Supplementary Material online). The sequence logo (top) compares a 67-amino-acid region spanning these domains with orthologous regions from other mammals. The sequence alignment (middle) compares human *IFITM3* with the most similar representative IR-IFITM ortholog from the other species (selected sites highlighted and shown in bold).

An important downstream consequence of activation of type I IFNs is the expression of various antiviral IFN-stimulated genes (ISGs) (de Weerd et al. 2007). In bats, several of these genes, such as *tetherin* and *APOBEC3*, have also been shown to be under positive selection (Hayward et al. 2020) or duplicated (Jebb et al. 2020). Among antiviral ISGs, we observed an expansion of the immune-related IFN-induced transmembrane (IR-IFITM) gene family (+1 gene; Viterbi method, *P* = 0.018) on the branch leading to the Yangochiroptera, the suborder that includes most microbats (fig. 2). The IR-IFITMs—which have previously been reported to be under positive selection in bats (Benfield et al. 2019)—are potent broad-spectrum antiviral factors that help to prevent infection before a virus passes the lipid bilayer of the cell (Bailey et al. 2014; Desai et al. 2014). By applying a branch-site likelihood ratio test (see Materials and Methods) based on the nonsynonymous/synonymous rate ratio (dN/dS) also known as ω, we confirmed using our data that the IR-IFITMs show evidence of positive selection in the bat MRCA (*P* = 6.4e−3). Furthermore, we identified seven particular codon sites that show signs of episodic diversifying selection (supplementary table S7, Supplementary Material online), including three sites in the CD225 domain (codons 68, 70, and 117). Notably, codons 68 and 70 in the CD255 domain are among several sites previously shown to be critical for blocking viral infection (Benfield et al. 2019). Furthermore, the methionine-to-phenylalanine substitution at codon 68 occurs in an amphipathic helix previously shown to be essential for blocking viral infection (Chesarino et al. 2017). Together, these observations of gene duplications and positive selection in functional domains in IR-IFITMs suggest that this gene family may have played an important role in the evolution of antiviral responses in bats.

### Expansion of *PRDM9* in Phyllostomid Bats and Expansion of Heat Shock Proteins in *P*. *mesoamericanus*

A third gene family to emerge from our survey of gene expansions and contractions was *PRDM9* (supplementary table S8, Supplementary Material online), which specifies the location of meiotic recombination sites (Paigen and Petkov 2018) and is known to evolve rapidly in vertebrates (Baker et al. 2017). *PRDM9* may play a role in speciation (Schwartz et al. 2014, 9) and is also upregulated upon viral infection (Xie et al. 2019). We found that *PRDM9* experienced a striking expansion in phyllostomid bats (+5 genes, Viterbi method *P* = 1.19e−05; supplementary fig. S5, Supplementary Material online), far beyond anything observed in other mammals. This observation has not, to the best of our knowledge, been made in the literature and was facilitated by our high-quality, long-read genomes. Our genomes for *P*. *mesoamericanus* and *A*. *jamaicensis* revealed that the expansion is specific to the phyllostomids and that a substantially larger expansion of *PRDM9* occurred in *A*. *jamaicensis* than can be observed in the short-read assembly (supplementary table S9, Supplementary Material online). Furthermore, comparison with a recent analysis of *PRDM9* in 446 vertebrates suggests that the highest *PRDM9* copy number is found in phyllostomid bats (Cavassim et al. 2022). Phyllostomid bats stand out for their morphological diversity and their extensive chromosomal rearrangements (Sotero-Caio et al. 2013), raising the possibility that the expansion of *PRDM9* and its effect on meiosis played a role in these traits. Intriguingly, many of the *PRDM9* copies in phyllostomid bats have lost the KRAB domain (supplementary table S9, Supplementary Material online), which is thought to play an important role in recruiting the recombination machinery (Baker et al. 2017), suggesting they may have alternate functions.

Finally, we observed a major expansion in *P*. *mesoamericanus* of heat-shock proteins across multiple gene families, including heat-shock protein 70 kDa (PTHR19375, +10 genes; Viterbi method, *P* = 8.9e−9). Interestingly, this expansion was largely restricted to *P*. *mesoamericanus* and was not observed across other bats. Overexpression of heat-shock proteins can modulate immune responses (Tsan and Gao 2009); therefore, this duplication may have implications for immunity in *P*. *mesoamericanus*.

### Positive Selection Analysis

**Fig. 3.**
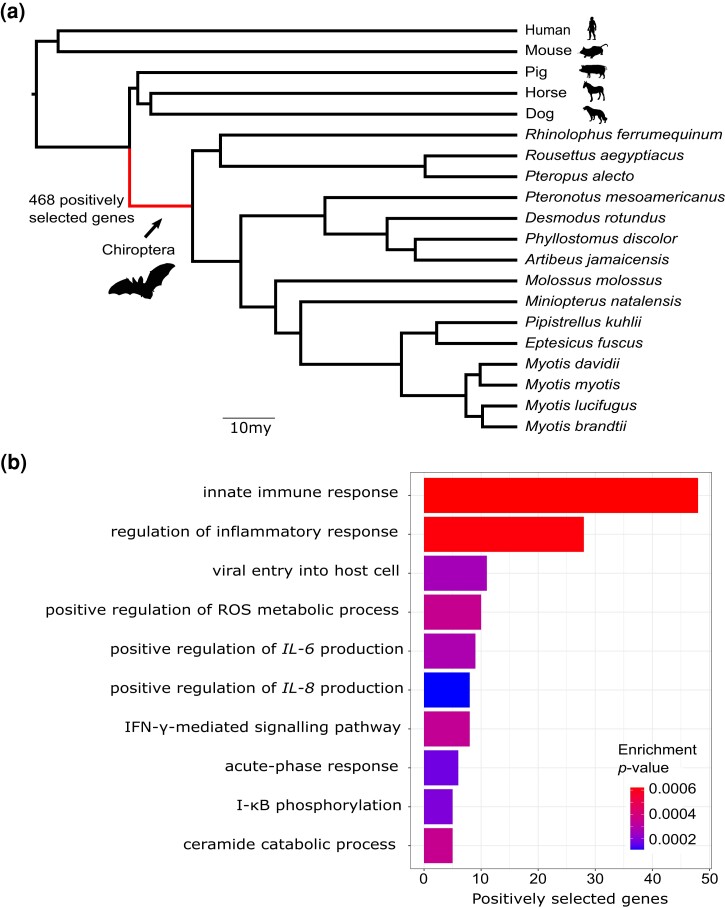
Positive selection scan on the bat ancestral branch. (*a*) Maximum likelihood phylogeny based on codon-site partitioned analysis of 3,632 gene alignments, with the bat ancestral branch indicated. (*b*) TopGO hierarchical GO enrichment analysis of the PSGs against the background set of tested genes, suggesting strong enrichment of innate immunity genes. The ten most significant GO terms are shown, eight of which are related to innate immunity including genes involved in interleukin regulation and IFN pathways.

Having identified signatures of positive selection at the amino-acid level in several gene families of interest, we applied similar branch-site likelihood ratio tests (Yang and Nielsen 2002; Zhang et al. 2005; Smith et al. 2015) genome wide, focusing on 12,517 single-copy orthologs present in bats and outgroup mammals. Because we were interested in molecular traits shared by most bats, we focused on a test for positive selection on the branch leading to the bat MRCA, where we expected to have reasonably good statistical power. However, we also tested for positive selection on the lineages leading to each of the two newly sequenced bats. Observing a highly skewed distribution of nominal *P*-values (*P* > 0.98 in 86% of tests) indicative of a misspecified null model, we opted to follow a recent study in bats (Potter et al. 2021) and omit a correction for multiple comparisons across orthologs, instead adjusting only for testing on three different branches (but see supplementary table S10 and supplementary Data S1, Supplementary Material online for more conservative adjusted *P*-values). As the simulation study conducted by Potter et al. (2021) shows, multiple testing correction of branch-site test *P*-values can be overly conservative and can remove substantial numbers of true positives. Based on our testing strategy, we identified 468 positively selected genes (PSGs) with an adjusted *P* < 0.05 in the bat MRCA (fig. 3; supplementary table S10, Supplementary Material online). This number is roughly comparable with the 298 PSGs recently identified on the phyllostomid bat lineage using a similar filtering approach (Potter et al. 2021), but somewhat larger than the 181 PSGs (Hawkins et al. 2019) and 23 PSGs (Jebb et al. 2020) found on the bat ancestral branch in recent studies that used more stringent criteria. These PSGs were strongly enriched for immune-related functions (supplementary table S11, Supplementary Material online), including the major gene ontology (GO) biological processes “regulation of inflammatory response” (GO:0050727, *P* = 6.1e−4) and “innate immune response” (GO:0045087, *P* = 6.2e−4). In total, 125 PSGs (27% of the 468) were annotated with the parent term “immune system process” (GO:0002376). The bat MRCA branch was also enriched for PSGs involved in “positive regulation of ROS” (GO:2000379, *P* = 3.5e−4), possibly suggesting adaptations associated with heightened metabolic rates owing to flight (see Discussion). Moreover, we detected 6 autophagy-related PSGs, including autophagy regulator *ATG9B* which was previously implicated in bat longevity (Kacprzyk et al. 2021). Below, we further discuss some specific PSGs falling in two major classes: 1) pathogen sensors, cytokines, and antiviral genes and 2) DNA repair genes and tumor suppressors.

### Pathogen Sensors, Interleukins, and Antiviral Genes in Bats Are Rapidly Evolving

The 468 PSGs on the bat ancestral branch included several genes that encode proteins with pathogen-sensing roles. For example, the TLR-encoding genes *TLR7* and *TLR8*, are included in our set (supplementary table S10, Supplementary Material online), and the related *TLR9* (which shows reduced activation in bats [Banerjee et al. 2017]) was on the threshold of statistical significance (*P* = 0.05). These genes have been identified in previous scans for positive selection (Escalera-Zamudio et al. 2015; Jiang et al. 2017). Another previously identified PSG in our set is the IFN stimulator *STING* (Xie et al. 2018). The TLRs and *STING*, as well as the PSG *NLRP3* (Zhang et al. 2013; Ahn et al. 2019), all play important roles in inflammation and are considered therapeutic targets for inflammatory disease (Hennessy et al. 2010; Fitzgerald and Kagan 2020; Gouravani et al. 2020; Decout et al. 2021). Positive selection in these genes may play a role in dampening downstream responses to pathogens. Interestingly, the location of a bat-specific substitution in codon 358 of *STING* that was linked to dampened IFN activation (Xie et al. 2018) was identified in our scan as one of ten positively selected sites (supplementary table S7, Supplementary Material online). In addition to these previously identified PSGs, we identified *TLR2* to be under positive selection. Unlike the nucleic-acid sensing TLRs 7, 8, and 9, TLR2 recognizes lipoproteins of pathogens such as bacteria and enveloped viruses (Oliveira-Nascimento et al. 2012). TLR2 signaling also induces the inflammatory cytokine TNF-α in infections with viruses like SARS-CoV-2, and blocking TLR2 protects against pathogenesis caused by the “cytokine storm” (Zheng et al. 2021). We additionally found evidence of positive selection in the key TLR regulator *UNC93B1* (Kim et al. 2008), which is regulated by type I IFNs (Panchanathan et al. 2013). UNC93B1 is thought to regulate nucleic-acid sensing TLRs such as TLR7 and TLR9 upstream of the process of TLR trafficking from the endoplasmic reticulum to endolysosomes (Lee et al. 2013; Pelka et al. 2018).

Another group prominently represented in our PSGs are genes encoding the interleukins, a collection of cytokines with diverse functions in immunity and inflammation (Garlanda et al. 2013; Veldhoen 2017). In bats, earlier work identified reduced expression of *interleukin-8* (Banerjee et al. 2017), positive selection of several interleukins and interleukin receptors (Moreno Santillán et al. 2021), and loss of *interleukin-36γ* (Jebb et al. 2020), suggesting that rapid evolution of interleukins may have contributed to unique molecular adaptations in bat immunity. We found that 5 interleukin-related GO categories were significantly enriched for PSGs in bats (supplementary table S11, Supplementary Material online), including “interleukin-1β production” (GO:0032611, *P* = 0.00075) and “positive regulation of interleukin-6 production” (GO:0032755, *P* = 0.00028). In addition, we identified the pleiotropic cytokine-encoding genes *interleukin-6* and *interleukin-15* as PSGs as well as the genes encoding several interleukin-associated receptors (supplementary table S10, Supplementary Material online). *Interleukin-6*, which encodes one of the most important cytokines during infection (Velazquez-Salinas et al. 2019), showed 6 sites predicted to be positively selected in bats and a high ratio of nonsynonymous substitutions per nonsynonymous site to the number of synonymous substitutions per synonymous site on the bat MRCA branch (dN/dS = 2.72). Inflammatory cytokine-encoding genes such as *interleukin-6* and *interleukin-15* that were under positive selection in the bat MRCA could be additional contributors to dampened inflammation in bats.

**Fig. 4.**
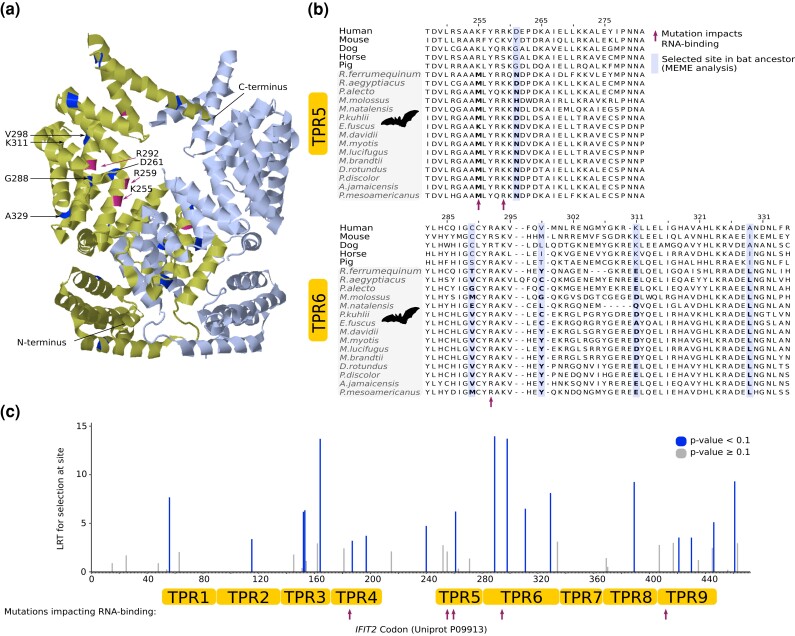
The antiviral gene *IFIT2* is positively selected in RNA-binding regions in bats. (a) 3D structure of the IFIT2 protein (PDB:4G1*T*) showing sites selected in the bat ancestor and sites known to be involved in RNA-binding function of the protein. (*b*) Amino acid alignment of the tetratricopeptide repeat (TPR) regions TPR5 and TPR6 (based on UniProt annotation for P09913) showing sites selected in the bat ancestor (highlighted and bold) and a fixed bat lysine-to-methionine substitution in codon 255 which is located in a region involved in RNA-binding (indicated with arrow). (*c*) MEME analysis of *IFIT2* codon sites showing 18 sites selected in bats based on a likelihood ratio test.

**Fig. 5.**
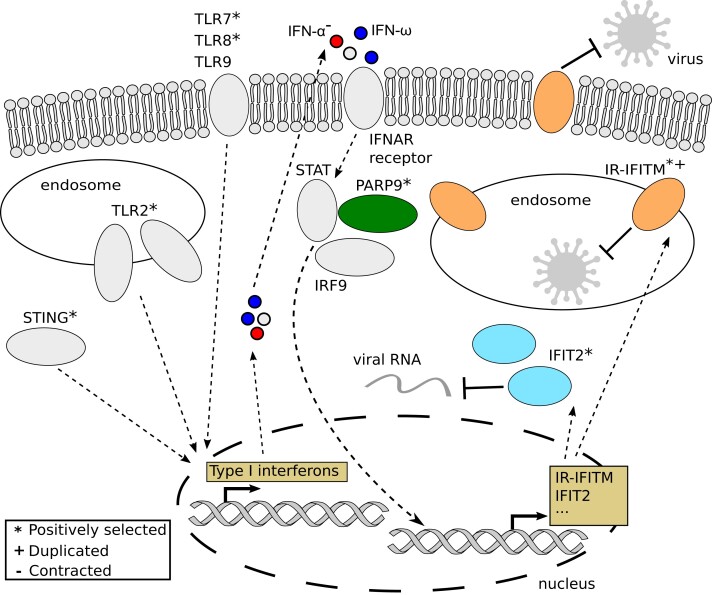
Schema of cellular innate immunity processes associated with genes rapidly evolving in bats. Proteins shown in color are the most significant innate immunity proteins highlighted in this study. Pathogen sensing pathways involving TLRs and *cGAS*-*STING* can induce expression of type I IFNs including IFN-α and IFN-ω. IFN-α genes were lost in the bat ancestor, potentially giving a greater role to IFN-ω. The type I IFNs trigger the induction of ISGs via pathways including *STAT*, which interacts with the positively selected *PARP9*. The ISGs include the positively selected *IFIT2* gene and the immune-related (IR) IFITM genes duplicated in Yangochiroptera bats, with both *IFIT2* and IR-IFITM genes playing prominent roles in antiviral defenses. The overall schema is based on reviews of type I IFNs, PARP9, IFIT, and IFITM proteins (Diamond and Farzan 2013; Zhu and Zheng 2021) and studies on IFITM interactions with RNA viruses (Brass et al. 2009; Foster et al. 2016).

Our PSGs also included several antiviral ISGs, which, as noted above, are activated by type I IFNs (de Weerd et al. 2007) and in several cases (including *APOBEC3* and *Mx*) have been shown to be duplicated (Jebb et al. 2020) and/or under positive selection (Fuchs et al. 2017; Hayward et al. 2018) in bats. For example, we detected positive selection in *PARP9*, whose gene product interacts with DTX3L and STAT1 to enhance IFN responsiveness (Zhang et al. 2015; Xing et al. 2021). We also found strong evidence of positive selection, affecting 18 codons, in *IFIT2*, whose product inhibits replication of a broad range of RNA and DNA viruses (Diamond and Farzan 2013; Fensterl and Sen 2014) (fig. 4). Ten of the positively selected sites overlap or are physically close to sites known to impact RNA-binding in the TPR4, TPR5, TPR6, and TPR9 motifs (Yang et al. 2012), and a bat-specific lysine-to-methionine substitution occurs at codon 255 which is involved in RNA-binding (Yang et al. 2012) (fig. 4). Positive selection of *IFIT2* in bats may therefore alter, and possibly enhance, expression of numerous antiviral response genes. Taken together, our results provide novel evidence for rapid evolution in the innate immune system of bats (fig. 5).

### DNA Repair Genes and Tumor Suppressors Are Positively Selected in Bats

Enhanced DNA repair has been proposed as a mechanism for longevity and cancer resistance in various mammals including bats (Seim et al. 2013; Tollis et al. 2017). We identified six DNA repair-related PSGs and 46 PSGs that were “cancer-related” (supplementary table S10, Supplementary Material online), meaning they were included in either the Tumor Suppressor Database (Zhao et al. 2016) or the Catalogue Of Somatic Mutations In Cancer (Forbes et al. 2011). Notably, cancer-related genes were enriched more than 2-fold among PSGs on the bat ancestral branch relative to a set of mammalian branches (Fisher's exact test: *P* = 9e−3, odds ratio = 2.2). Among DNA repair genes, we detected evidence of positive selection in the tumor suppressor-encoding *PALB2* and in four DNA polymerase-encoding genes (*POLA1*, *POLD1*, *POLK,* and *POLM*). *PALB2* is a crucial component of the BRCA complex and is required for homologous recombination repair (Sy et al. 2009; Deveryshetty et al. 2019; Belotserkovskaya et al. 2020). In bats, it shows three sites under selection as well as seven bat-specific coding indels, including a 21-nucleotide deletion in a RAD51/BRCA1-interacting region (Uniprot annotation: Q86YC2). Despite previous evidence in a long-lived bat (Zhang et al. 2013), we did not find a signal for selection in *BRCA2*, although it does contain 14 bat-specific indels. Similarly, we did not identify the DNA repair genes *RAD50* and *KU80* (Zhang et al. 2013) or the key tumor suppressor gene *TP53* as PSGs, but we did find four sites (codons 35, 38, 54, 97) in *TP53* that are potentially selected in bats as well as a previously described bat-specific indel in the nuclear localization signal domain (Zhang et al. 2013) (supplementary fig. S6, Supplementary Material online).

Although *TP53* did not appear among our PSGs, we did identify genes encoding two other tumor suppressors that interact with it (table 1): *BCL-2 interacting killer* (*BIK*) and *large tumor suppressor kinase* (*LATS2*). Both genes showed highly significant signals of selection in bats in our data set but have not been identified in tests of other mammalian branches (Nielsen et al. 2005; Kosiol et al. 2008) or in earlier studies in bats (Hawkins et al. 2019; Jebb et al. 2020). LATS2 is a kinase that modulates the functions of tumor suppressors such as TP53 as well as canonical growth-related Hippo signaling effectors YAP/TAZ (Furth and Aylon 2017). We found that *LATS2* is predominantly under negative selection (bat ancestor ω = 0.23, outgroup mammals ω = 0.11) but nevertheless contains 13 nonsynonymous substitutions in bats as well as seven bat-specific microindels in its coding region (supplementary fig. S7 and supplementary Data S2, Supplementary Material online). Four of the substitutions fall within known functional domains of the protein, with a codon 134 glutamic-acid-to-aspartic-acid substitution in the LATS conserved domain 1 (LCD1) predicted to have an effect on the protein based on SNPeffect (Reumers et al. 2005). Previous experimental work in mice has shown that the LCD1 domain is critical for tumor suppressor activity of LATS2 (Yu et al. 2015). The second tumor suppressor, BIK, belongs to the proapoptotic BH3-only family of proteins that are upregulated in response to various stress signals and act as antagonists of prosurvival proteins (Huang and Strasser 2000; Zou et al. 2002). BIK is regulated by TP53 (Hur et al. 2006; López et al. 2017) and contributes to the apoptotic response induced by chemotherapy treatments (Zou et al. 2002; Real et al. 2006). The rapid molecular evolution of *LATS2* and *BIK* suggests that these genes may contribute to bat molecular adaptations in tumor suppression.

**Table 1:** Positively Selected Genes Involved in Cancer (*P* < 1.0e−3 with At Least One Site Selected in the Bat MRCA) that Showed the Strongest Statistical Significance. The *P*-Values Shown (aBSREL P-Value) are Derived from the Branch-Site Likelihood Ratio Test and Adjusted for Multiple Comparisons Across Branches but not Across Genes (see Text). Values of ω > 10 can Occur Due to Limited Signal and Imply Uncertain Estimates. Sites with Evidence of Positive Selection in the Bat MRCA were Identified Using Site-Wise MEME Analysis with a Significance Threshold of *P* < 0.1

Symbol	Name	aBSREL *P*-Value	Sites under Selection	Bat Branch ω	Outgroup Branch ω
*CDH1*	*Cadherin-1*	2.0e−6	3	0.17	0.29
*CAT*	*Catalase*	7.0e−6	1	0.38	>10
*BIK*	*BCL2 interacting killer*	3.7e−5	1	1.92	0.89
*PALB2*	*Partner and localizer of BRCA2*	1.8e−4	3	0.67	0.43
*LATS2*	*Large tumor suppressor kinase 2*	2.5e−4	10	0.23	0.11
*SLC39A4*	*Solute carrier family 39 member 4*	3.8e−4	3	0.55	0.42
*SPARCL1*	*SPARC like 1*	4.0e−4	4	0.88	0.95
*PLA2G7*	*Phospholipase A2 group VII*	8.5e−4	5	>10	1.70
*GALR1*	*Galanin receptor 1*	8.7e−4	1	0.10	0.08
*CD79A*	*CD79a molecule*	9.0e−4	1	>10	0.37
*MYO1A*	*Myosin IA*	9.8e−4	7	0.94	0.14

The two cancer-related genes displaying the strongest signals of positive selection, *cadherin-1* (*CDH1*) and *catalase* (*CAT*) (table 1), also play roles in tumor development. CDH1 belongs to a family of transmembrane glycoproteins that mediate cell–cell adhesion and regulate cell growth, making them therapeutic targets for preventing tumor progression (Yu et al. 2019). CAT is an antioxidant enzyme that can protect from ROS-induced stress (Glorieux and Calderon 2017) and plays a role in TP53-mediated ROS regulation in response to DNA damage (Kang et al. 2013, 53). Although these functions make *CAT* and *CDH1* intriguing examples of genes that have potentially evolved to enhance bat cancer resistance, previous studies have also found a signal for selection in *CAT* (Enard et al. 2016; Slodkowicz and Goldman 2020) and *CDH1* (Enard et al. 2016; Vicens and Posada 2018; Slodkowicz and Goldman 2020) in other mammals. Experimental validation of the bat-specific mutations detected here will thus be important to demonstrate their potential adaptive value.

## Discussion

High-quality and complete genome sequences are indispensable for revealing patterns of genomic variation within and between species. In this study, we used long-read sequencing to assemble the genomes of the bats *A*. *jamaicensis* and *P*. *mesoamericanus* and analyzed them together with 13 additional bat genomes to provide insights into the unique molecular evolution of bats. We found evidence of positive selection and structural variation in three key components of the bat innate immune system, including pathogen sensing, type I IFN cytokine signaling, and IFN-stimulated antiviral genes (see schema in fig. 5). Our results highlight how bat immune systems rely on both bat-wide and lineage-specific evolution in the immune gene repertoire, suggesting an interplay of diverse immune strategies with core molecular adaptations that occurred in the bat ancestor such as the loss of the PYHIN inflammatory gene family. In particular, the loss of IFN-α genes and the potentially increased reliance of bats on IFN-ω may play a role in their tolerance of viral infections. The expansion of antiviral IR-IFITM genes and *PRDM9* as well as APOBEC3 (Jebb et al. 2020) and MHC-I (Moreno Santillán et al. 2021) class genes in major bat clades may have further contributed to lineage-specific evolution. Our findings underline the rapid evolution of the bat innate immune system, which has also been shown to have undergone additional gene losses in pathways related to defensins, natural killer signaling, and IFNs (Ahn et al. 2016; Jebb et al. 2020; Moreno Santillán et al. 2021). Thus, a combination of expansions of antiviral genes and losses of proinflammatory genes may contribute to the dampened inflammatory response and viral tolerance in bats. We also found evidence of positive selection in the bat MRCA in 46 cancer-related genes, suggesting a possible link to the unusually low incidence of cancer in bats.

Perhaps our most striking finding, building on earlier comparative genomic studies of the complex type I IFN locus (Zhou et al. 2016; Pavlovich et al. 2018), is that nine type I IFNs were lost in the bat MRCA. Notably, we found that bats have lost most or—in the case of *Pipistrellus kuhlii*, *Myotis myotis*, and *P. mesoamericanus*—all of their IFN-α genes, making their type I IFN locus particularly distinct among mammals. The overall contraction of the type I IFN locus in bats has been hypothesized to allow a smaller number of constitutively expressed IFN-α genes to perform the functions of the 13 IFN-α genes in humans (Zhou et al. 2016). The constitutive expression and rapid evolution of bat IFNs may also partly result from dampened inflammation caused by evolutionary changes to inflammosome genes such as *AIM2*, *caspase-1* and *IL-1β* (Goh et al. 2020), because inflammosomes may negatively regulate type I IFN sensors (Yu et al. 2018). Indeed, the regulation of type I IFNs in bats also appears to have evolved adaptively, as positive selection of the key transcription factor *IRF3* in bats was previously shown to enhance antiviral responses via type I IFN activation (Banerjee et al. 2020). However, our results suggest that constitutive expression of IFN-α is not common to all bats, in line with expression analyses in *R*. *aegyptiacus* (Pavlovich et al. 2018). We hypothesize that by relying on the potentially more potent IFN-ω rather than IFN-α, bats may further enhance their antiviral responses. Although further work will be needed to demonstrate a functional shift to IFN-ω, the lack of any IFN-α genes in three bat species strongly suggests a shift has occurred at least in these cases. It is possible that an enhanced antiviral response owing to IFN-ω helps to compensate for an overall dampened inflammatory response in bats. If these properties of IFN-ω can be established, they may open the door to new therapeutic uses of IFN-ω (Zhao et al. 2009; Li et al. 2017).

In addition, our findings suggest that, compared with other parts of the mammalian phylogeny, the lineage leading to bats is enriched for positively selected cancer-related genes. Rapid evolution of DNA repair genes as well as tumor suppressors has been proposed as a mechanism for cancer resistance in other long-lived mammals such as whales (Tejada-Martinez et al. 2021). Intriguingly, it was previously hypothesized that selection of cancer resistance components such DNA repair genes in bats resulted from a need to reduce the negative effects of ROS generated as a consequence of flight (Zhang et al. 2013). This suggestion appears consistent with our findings of positively selected ROS regulators and DNA repair genes. The evolution of cancer resistance in bats may also be associated with adaptations in the bat immune system. There is substantial overlap of genes related to cancer and the immune system (de Fonseca et al. 2010), with immune-related genes being known to play a role in cancer surveillance (Ostrand-Rosenberg 2008) and tumor suppression (Li et al. 2011; Siegrist et al. 2011; Alteber et al. 2018). Specific examples include immune-related genes that are rapidly evolving in bats. For example, ROS plays an important role in *NLRP3* inflammasome activation (Moossavi et al. 2018; Yang et al. 2019), *IFIT2* plays a role in apoptosis and cancer progression (Pidugu et al. 2019), and IFNs contribute to antitumor activity (Aricò et al. 2019; Lu et al. 2019). Positive selection in such genes may be driven by fitness trade-offs between roles in immunity and cancer (de Fonseca et al. 2010). For example, inhibition of inflammation can promote longevity (Youm et al. 2013; Marín-Aguilar et al. 2020) and suppress tumor growth (Lee et al. 2019; Tengesdal et al. 2021). Comparative analyses of gene expression across mammals and experimental validation may help resolve the different roles of ROS regulation, DNA repair, inflammation, and immunity in cancer resistance. We anticipate that our evolutionary findings and the novel genomic resources we have made available (including a genome browser) will encourage and facilitate further genomic research in bats, particularly as models that can lead to new strategies for addressing major challenges to human health such as infectious diseases and cancer.

## Materials and Methods

### Sample Background and Acquisition

Fresh liver samples from a single individual of *A. jamaicensis* (AMNH.Mammals.279493, male) and one *P. mesoamericanus* (AMNH.Mammals.279536, male) were collected by N.B.S. in April 2017 at the Lamanai Archaeological Reserve in Orange Walk District, Belize (17.75117°N, 88.65446°W). Sampling followed best practices for humane capture, handling, and euthanasia of live mammals outlined by the American Society of Mammalogists (Sikes 2016). All work was conducted with permission of the Belize Forest Department under permit number WL/2/1/17 (19) with Institutional Animal Care and Use Committee (IACUC) approval from the American Museum of Natural History (AMNHIACUC-20170403) and University of Georgia (A2014 04-016-Y3-A5). Bats were captured in ground-level mist nets and placed in individual cloth bags for transport to the Lamanai Field Research Center. After identification, the bats were euthanized using isoflurane, and the liver was removed immediately after death. Samples were placed in multiple individual 2 ml cryotubes and flash-frozen by placement in a liquid nitrogen dry shipper. The cold chain was maintained through shipment to the AMNH, storage in the AMNH Ambrose Monell Cryo Collection, and subsequent sample processing and transfers.

### Genome Sequencing and Assembly

Approximately 40 mg of liver tissue from each bat was received at Cold Spring Harbor Laboratory (CSHL) and stored at −8 °C. The liver samples were crushed with a micro pestle and mixed with 10 ml of TLB buffer and 50 μl of RNase A immediately before use. After 1-h incubation at 37°C, 50 μl of proteinase K was added, followed by incubation at 5 °C for 3 h with hourly inversion mixing. After addition of 10 ml of a phenol chloroform/isoamyl alcohol mixture, each sample was rocked for 10 min and then centrifuged at 4500 RPM for 10 min. The top aqueous layer was retained and an equal volume of chloroform/isoamyl alcohol was added, and rocking and centrifugation were repeated as above. The top layer was transferred to a fresh tube with 4 ml of 5 M ammonium acetate. Following the addition of 30 ml of ice cold 100% ethanol, the sample was rocked for 10 min. The visible DNA was then extracted with a glass pipet and placed in a 1.5 ml tube. The sample was washed once with 100% ethanol and centrifuged for 5 min at 10,000 RPM. The ethanol was removed, and any remaining ethanol was evaporated on a 37 °C heat block for 10 min. The final DNA was resuspended in 10 mM Tris HCl pH 8.5 and stored overnight at 4 °C.

DNA was then sheared to ∼50–75 kb using a Diagnode Megarupter following manufacturer's recommendations. DNA was further enriched for long fragments via the Circulomics small read eliminator XL kit, which iteratively degrades short fragments. DNA was prepared for Nanopore sequencing using the ONT 1D sequencing by ligation kit (SQK-LSK109). Briefly, 1–1.5 μg of fragmented DNA was repaired with the NEB FFPE repair kit, followed by end repair and A-tailing with the NEB Ultra II end-prep kit. After an Ampure clean-up step, prepared fragments were ligated to ONT specific adapters via the NEB blunt/TA master mix kit. The library underwent a final clean-up and was loaded onto a PromethION PRO0002 flowcell per manufacturer's instructions. The flowcells were sequenced with standard parameters for 3 days. Basecalling was performed in real time with Guppy 3.2 and reads were later re-basecalled with Guppy 4 to improve quality. Nanopore reads were filtered for minimum length of 10 kb and minimum 85% accuracy using filtLong 0.2.0 (http://github.com/rrwick/Filtlong). The resulting coverage was 96.11× for *A*. *jamaicensis* (read N50 of 25.47 kb and maximum read length of 815.21 kb) and 99.19× for *P*. *mesoamericanus* (read N50 of 26.01 kb and maximum read length of 1.68 Mb).

Illumina short read libraries were prepared from the same tissue as above with the Illumina TruSeq DNA kit, targeting a 550 bp insert size with polymerase chain reaction enrichment. Libraries were sequenced at the New York Genome Center, on a NovaSeq S4 flowcell in a paired-end 150 bp format to ∼30× genome coverage. Short reads were used only for polishing, not assembly.

Reads were assembled using flye 2.8.3 (Kolmogorov et al. 2019) with “–nano-raw –no-alt-contigs” flags, after evaluation of additional assemblies generated with wtbg2 2.5 (Ruan and Li 2020), NextDenovo 2.2 (Hu et al. 2023), and Shasta 0.7.0 (Shafin et al. 2020) (supplementary table S12, Supplementary Material online). Assembly was followed by one round of long-read polishing using minimap 2.17 (Li 2018) with default parameters for alignment and PEPPER 0.1 (Shafin et al. 2021) with the “PromethION_r941_guppy305_HAC_human.pkl” model for polishing. Next, bwa-mem 0.7.17 (Li 2013) was used to align the Illumina short-read data to the long-read polished assembly with default parameters, and one round of short-read polishing was carried out with POLCA from (Zimin and Salzberg 2020) from MaSuRCA 3.4.0 using default parameters. To compare assembly contiguity, error rates, and completeness, assemblies were then assessed using Merqury 1.0 (Rhie et al. 2020), as well as with BUSCO 4.0.5 (Simão et al. 2015) for mammals (odb9). Additionally, we used python scripts to compute the cumulative sum of contigs length (N [X] length) versus the cumulative sum of N (X)% of the total genome. Further assembly statistics were calculated using BBTools 38.86 (http://sourceforge.net/projects/bbmap/). Duplicated haplotypes in the assemblies were purged using purge_dups 1.2.3 (Guan et al. 2020).

The assemblies were aligned to human, mouse, and pig assemblies as well as to four bat assemblies (*M*. *myotis*, *Phyllostomus discolor*, *Desmodus rotundus,* and *Rhinolophus ferrumequinum*) using Cactus 1.0.0 (Armstrong et al. 2020). Bat-specific small indels (<1 kb) were called relative to the human reference from the multiple alignment using a custom python script. Indels were called in alignment blocks with at least ten bases and seven species aligned including at least two nonbat mammals. Only fixed indels present in all bats or all bats but one were retained. Bat-specific insertions and deletions relative to the human reference were distinguished based on the sequence of the pig outgroup.

### Gene Annotation

Public RNA-seq data from SRA were downloaded for *A. jamaicensis* and *Pteronotus parnelli* (supplementary table S13, Supplementary Material online), and proteins for human (GCF_000001405.39), mouse (GCF_000001635.26), and seven bat species (*Myotis brandtii*, GCF_000412655.1; *Myotis davidii*, GCF_000327345.1; *M. lucifugus*, GCF_000147115.1; *P. discolor*, GCF_004126475.1; *P. alecto*, GCF_000325575.1; *Rhinolophus ferumequinum*, GCF_004115265.1; and *R. aegyptiacus*, GCF_001466805.2) were downloaded from RefSeq. RNA-seq reads were aligned to the new reference genomes using HISAT 2.2.0 (Kim et al. 2019) with the parameters “–no-mixed –no-discordant –downstream-transcriptome-assembly”. Transcripts were assembled using StringTie 2.1.1 (Kovaka et al. 2019). To reduce potential loss of transcripts due to limitations of short-read alignment, transcriptomes were also de novo assembled with Trinity 2.9.1 (Grabherr et al. 2011) using default parameters. PASA 2.4.1 (Haas et al. 2003) was used to generate the final set of transcripts based on alignment with GMAP (Wu and Watanabe 2005) and BLAT (Kent 2002) using minimum thresholds of 90% of transcript length aligned at 90% identity. GeMoMa was used to project gene annotations from six bat assemblies (Jebb et al. 2020) to the genomes of *A*. *jamaicensis* and *P*. *mesoamericanus* with default parameters. Transdecoder with default parameters was used to predict coding sequences within the assembled transcripts (Haas et al. 2013). We used the transcripts and proteins sequences as evidence for the MAKER3 annotation pipeline (Cantarel et al. 2008) with the ab initio gene predictors SNAP 2006.07.28 (Korf 2004) and Augustus 3.3.3 (Stanke et al. 2006). GlimmerHMM (Majoros et al. 2004) and GeneMark 4.68 (Lomsadze et al. 2005) were used to generate further ab initio gene predictions. Final annotations were generated with EVidenceModeler 1.1.1 (Haas et al. 2008), weighting ab initio and MAKER predictions as 1, protein alignment evidence as 2, transcript evidence as 5, and PASA evidence as 10. To reduce the number of potentially missing genes caused by lack of protein and RNA-seq evidence, we used intact TOGA 1.0.0 (Kirilenko et al. 2023) gene projections from the recently assembled *A*. *jamaicensis* (GCF_014825515.1) to add gene annotations that did not intersect the Evidence Modeler annotations. The completeness of the final predicted protein set was assessed using BUSCO.

### Repeat Analysis

Repeat masking was carried out using an iterative masking and de novo repeat detection approach. After masking repeats with RepeatMasker 4.0.9 (Tarailo-Graovac and Chen 2009) and the combined RepBase-20181026 and Dfam-3.1 repeat databases, novel repeats were detected in the masked genomes with RepeatModeler 2.0.1 using default parameters. The consensus de novo repeats longer than 100 bp were then concatenated with a vertebrate repeat library including novel bat repeats (Jebb et al. 2020) and clustered with CD-HIT 4.81 (Fu et al. 2012). All clustered novel sequences with >80% sequence similarity across >80% of the length of the clustered sequences were excluded (Wicker et al. 2007). Novel repeats were then aligned to the nt database using BLAST+ 2.7.1, and all repeats matching annotated transcripts were removed. A further BLAST analysis was used to exclude repeats with fewer than 10 alignments to the reference genome from which they were derived. Transposable elements were classified and assigned families using TEclass (Abrusán et al. 2009) and DeepTE (Yan et al. 2020). The consensus de novo repeats were then concatenated with the vertebrate repeat library, and a final masking of the genome was carried out with RepeatMasker using the “sensitive” setting. The recently diverged repeat landscape was analyzed using the RepeatMasker script calcDivergenceFromAlign.pl with correction of substitution rates based on the Kimura 2-Parameter model. Transposons were considered recently diverged at 7% divergence from the consensus (Jebb et al. 2020), which approximates an insertion <30 Ma, assuming a mammalian substitution rate of 2.2 × 10^−9^ (Kumar and Subramanian 2002). To allow comparison of the repeat landscape within Noctilionoidea bat clade, the same repeat masking approach was carried out for several closely related bats (*D. rotundus*, *P. discolor*, *Sturnira hondurensis*, and *M*. *myotis*).

### Mining of Endogenous Viral Elements

To scan for endogenous viral elements that might provide evidence of past infections, we aligned the complete RVDB-prot viral protein database (Bigot et al. 2019) to our genomes using BLAST with an e-value threshold of 1e−5. Alignments intersecting coding sequence and those shorter than 100aa were excluded. Nonretroviral matches were aligned to the NR protein database using blastx with an e-value threshold of 1e−5 and TaxonKit 0.6.0 (Shen and Xiong 2019) was used to retain sequences with a best match to a viral lineage.

### Identification of Orthologous Gene Groups

We analyzed bat genes for lineage-specific signals of positive selection and gene duplications based on clustered ortholog groups. Coding sequences for the seven bats used for annotation and six outgroup mammals (human, mouse, dog, pig, and horse) were downloaded from RefSeq. Annotated open reading frames that did not show a nucleotide number that was a multiple of three or that contained internal stop codons were discarded. The longest isoform for each gene was retained and translated to an amino acid sequence using Biopython (Cock et al. 2009). All proteins were clustered with the proteins of our bats using OrthoFinder 2.3.11 (Emms and Kelly 2019). A set of single copy orthologs was extracted from the OrthoFinder results, retaining orthologs with at least 12 bat species and three mammalian outgroups. Genes were then aligned using PRANK v.170427 (Löytynoja 2014) with the species tree provided to guide the alignment. Cancer-associated ortholog clusters were identified based on the databases TSG (Zhao et al. 2016) and COSMIC (Forbes et al. 2011).

### Gene Family Expansion Analysis

Alignments of 3,632 single copy genes that were present in all species were concatenated into a single alignment, which was divided into three partitions corresponding to codon positions. A maximum-likelihood phylogeny was inferred using RAxML 8.2.12 (Stamatakis 2014) rapid bootstrapping under the GTR + G + I model with 100 bootstraps. A dated phylogeny was then generated using MCMCtree in PAML 4.9 (Zhang et al. 2005). Node ages were calibrated based on TimeTree (Kumar et al. 2017) ages for Euarchontoglires, bats, Yangochiroptera, and Yinpterochiroptera. Convergence was assessed based on analysis of two replicate runs with tracer (Rambaut et al. 2018). The dated phylogeny was used to calculate gene family expansions and contractions with CAFE 5 (De Bie et al. 2006) based on a *P*-value threshold of 0.05. CAFE was executed using the base model with an error model (1.7% of gene families were estimated to have an error in gene size) and three λ values (gene birth–death rates) for the lineages Yinpterochiroptera, Yangochiroptera, and nonbat mammals. Assessment using likelihood ratio tests showed that two λ values (one for bats and one for nonbats) were a significantly better fit than one (*P* < 0.01) and three λ values were a significantly better fit than two (*P* < 0.01). Orthofinder orthogroups were collapsed into 7,405 PANTHER gene families based on BiomaRt 2.46.3 (Durinck et al. 2005) data. For each of 19,935 orthogroups, a representative gene was selected to obtain a PANTHER assignment. For 2,466 orthogroups, where no human or mouse gene was represented, we selected a gene for sequence-based annotation using interproscan 5.50 (Blum et al. 2021) and eggNOG 2.1.3 (Cantalapiedra et al. 2021). As CAFE assumes all genes were present in the common ancestor of all analyzed species, orthogroups represented in less than two bats and two outgroup mammals were excluded. Final copy numbers and genomic coordinates for IFITM (supplementary table S14, Supplementary Material online) and type I IFN (supplementary table S15, Supplementary Material online) genes were visualized using genoPlotR 0.8.11 (Guy et al. 2010). For the analysis of the *PRDM9* expansion in phyllostomids, recently generated short-read assemblies of *S. hondurensis* (GCA_014824575.2) and *A*. *jamaicensis* (GCA_014825515.1) were included in the phylogenetic analysis (supplementary fig. S5, Supplementary Material online) but not the CAFE analysis. Sequences and RAxML phylogenies are provided for IFITM, IFN, and *PRDM9* orthogroups (supplementary Data S3, Supplementary Material online). Silhouettes of species for our figures were obtained from PhyloPic (https://www.phylopic.org/).

### Positive Selection Analysis

We aimed to determine whether each gene was positively selected in one or more of three groups: the bat MRCA, *P*. *mesoamericanus*, and *A*. *jamaicensis*. We detected positive selection using the adaptive branch-site random effects model (aBSREL) method implemented in HyPhy 2.5.12 (Kosakovsky Pond et al. 2019). A likelihood ratio test was used to determine whether a lineage-specific group of codons in the alignment is experiencing significant positive selection. Unlike the branch site tests implemented in PAML (Zhang et al. 2005), aBSREL allows rate variation among the background branches, which can reduce false positive errors (Smith et al. 2015). In addition, we also used PAML to apply Test 2, a branch site test of positive selection. This test compares the alternative model where some branches are under positive selection and thus exhibit sites with ω > 1 with the corresponding null model where ω is fixed as 1. We computed *P*-values according to a χ^2^ distribution with one degree of freedom. Although accounting for nonadaptive processes when inferring selection in population-level analyses can be critical (Johri et al. 2022, 2023), branch site tests are well-powered to discriminate positive selection from negative or relaxed selection with low false positive rates in phylogenetic-scale analyses (Anisimova et al. 2002; Zhang et al. 2005; Yang and dos Reis 2011; Smith et al. 2015).

The *P*-values calculated by PAML and HyPhy were adjusted for multiple testing of three branches per gene using the Benjamini–Hochberg method for controlling false discovery rate (FDR) implemented in base R. We also corrected for multiple testing of all genes using FDR (supplementary table S10, Supplementary Material online). We used the maximum-likelihood species tree inferred using 3,048 orthologs (see Materials and Methods section “Gene Family Expansion Analysis”) to provide the topology for the positive selection scans. In this tree, bats are the sister group to Fereungulata (Cetartiodactyla, Perissodactyla, Carnivora, and Pholidota) (Jebb et al. 2020). Relationships within Fereungulata remain challenging to resolve (Doronina et al. 2017; Jebb et al. 2020), and here, we followed the TimeTree topology that places Perissodactyla + Carnivora as sister to Cetartiodactyla. For comparison with a previous scan for positive selection on all branches of a mammalian phylogeny (including human, chimpanzee, macaque, mouse, rat, and dog), we used the set of 8,594 genes that were tested for positive selection using PAML in both this study and in (Kosiol et al. 2008). MEME (Murrell et al. 2012) analysis was used to identify sites potentially under selection in the bat MRCA with a significance threshold of *P* < 0.1. Alignment of genes positively selected in the bat MRCA is provided in supplementary Data S4, Supplementary Material online.

### Gene Ontology Enrichment

GO, Reactome, and Kyoto Encyclopedia of Genes and Genomes (KEGG) annotations for genes were obtained via BiomaRt. A total of 18,452 orthogroups were annotated with at least one feature and 17,840 were assigned GO features. We carried out enrichment analysis on groups of genes that were positively selected or that showed gene family expansions. Enrichment analysis was performed using topGO 2.42.0, with the elim algorithm and Fisher's exact test (*P* < 0.01). All genes tested for selection were used as the background. The elim algorithm is a conservative approach that processes the GO graph from the bottom up, to eliminate higher order terms that would otherwise appear enriched due to correlation with more specific terms. GO terms with fewer than ten genes annotated were excluded.

## Supplementary Material

Supplementary data are available at *Genome Biology and Evolution* online (http://www.gbe.oxfordjournals.org/).

## Supplementary Material

evad148_Supplementary_Data

## Data Availability

The raw sequencing data and final genome assemblies generated in this study have been submitted to the NCBI BioProject database under accession number PRJNA751559. Gene annotations can be downloaded or viewed as UCSC genome browser tracks from our laboratory website (http://compgen.cshl.edu/bat/). The data underlying this article are available in figshare at https://doi.org/10.6084/m9.figshare.15223014.v3 (Scheben 2021). Code is available on GitHub at https://github.com/CshlSiepelLab/bat_genome_analysis.
