# Supplementary material for: Long-Read Sequencing Reveals Rapid Evolution of Immunity- and Cancer-Related Genes in Bats
Source: Genome Biol Evol. 2023 Sep 20;15(9):evad148. doi: 10.1093/gbe/evad148 (PMC10510315; doi:10.1093/gbe/evad148)
Supplement: evad148_Supplementary_Data [file evad148_supplementary_data.zip › Supplementary material legends.docx]

Supplementary table legends

**Table S1**. GenBank assemblies for the taxonomic order Chiroptera (bats).

**Table S2**. Comparison of assembly statistics of the unscaffolded genomes of *Artibeus jamaicensis* and *Pteronotus mesoamericanus* with five scaffolded bat genomes assembled using a range of technologies.

**Table S3**. Recently diverged repetitive elements in the genomes of five noctilionid bats and the related bat Myotis myotis.

**Table S4**. Non-retroviral endogenous viral elements (EVEs) detected in the genomes of *Artibeus jamaicensis* and *Pteronotus mesoamericanus*.

**Table S5**. Significantly expanded or contracted gene families in the bat lineage detected using CAFE.

**Table S6**. Fisher’s exact test using a 2-by-2 contingency table for IFN-ɑ and IFN-ω copy number in a set of outgroup mammals and in individual bat species.

**Table S7**. Significantly selected sites in the genes IFITM3, IFIT2 and STING in the bat lineage based on MEME analysis.

**Table S8**. Significantly expanded or contracted gene families in the bat subclade Yangochiroptera and in the species *Artibeus jamaicensis* and *Pteronotus mesoamericanus* detected using CAFE.

**Table S9**. Domain analysis of all protein sequences in the PRDM9 ortholog cluster for seventeen bats and four other mammals.

**Table S10**. Positively selected single copy orthologs in bats (order Chiroptera).

**Table S11**. TopGO gene ontology enrichment analysis of positively selected genes in the bat ancestor.

**Table S12**. Comparison of assembly and polishing statistics using different approaches.

**Table S13**. SRA accession numbers for RNAseq data used in the annotation of *Artibeus jamaicensis* and *Pteronotus mesoamericanus*.

**Table S14**. Genomic coordinates of the genes at the IFITM locus in nine bats and five other mammals.

**Table S15**. Genomic coordinates of the genes at the type I interferon locus in 10 bats and five other mammals.

Supplementary figure legends

**Figure S1**. Photographs of the bats *Artibeus jamaicensis* and *Pteronotus mesoamericanus* sequenced in this study

**Figure S2**. Genome quality statistics and repeat analysis.

**Figure S3**. Maximum-likelihood phylogeny of mammalian IFN-ɑ and IFN-ω genes showing the shift in the ratio of IFN-ɑ to IFN-ω copy number in bats compared to other mammals.

**Figure S4**. Comparison of the IFN locus in the *Artibeus jamaicensis* long read assembly with a previous short-read assembly based on Illumina reads and 10X data (GenBank accession: GCF_014825515.1)

**Figure S5**. Maximum likelihood phylogeny of the PRDM9 orthogroup generated using RAxML under the GTRGAMMA model.

**Figure S6**. Multiple alignment of *TP53* showing a bat-specific deletion (codon 320) in the nuclear localization signal domain.

**Figure S7**. Candidate sites under selection in bats in the *LATS2* gene.
