# Supplementary material for: Long-Read Sequencing Reveals Rapid Evolution of Immunity- and Cancer-Related Genes in Bats
Source: Genome Biol Evol. 2023 Sep 20;15(9):evad148. doi: 10.1093/gbe/evad148 (PMC10510315; doi:10.1093/gbe/evad148)
Supplement: evad148_Supplementary_Data [file evad148_supplementary_data.zip › Supplementary_Figures.pdf]

Supplementary figures

Long-read sequencing reveals rapid evolution of immunity- and cancer-related genes in bats

Armin Scheben[1], Olivia Mendivil Ramos[2,#], Melissa Kramer[2], Sara Goodwin[2], Sara Oppenheim[3], Daniel J Becker[4], Michael C Schatz[1,5], Nancy B Simmons[6], Adam Siepel[1*], W Richard McCombie[2*]

[1] Simons Center for Quantitative Biology, Cold Spring Harbor Laboratory, Cold Spring Harbor, NY
[2] Cold Spring Harbor Laboratory, Cold Spring Harbor, NY
[3] American Museum of Natural History, Sackler Institute for Comparative Genomics, New York, NY
[4] Department of Biology, University of Oklahoma, Norman, OK
[5] Departments of Computer Science and Biology, Johns Hopkins University, Baltimore, MD
[6] Department of Mammalogy, Division of Vertebrate Zoology, American Museum of Natural History, New York, NY
[#] Current address: OneThree Biotech, 335 Madison Ave, New York, NY

Table of Contents

**Supplementary Figures**	
**Figure number**	**Figure title**
**Figure S1**	Photographs of the bats *Artibeus jamaicensis* and *Pteronotus mesoamericanus* sequenced in this study
**Figure S2**	Genome quality statistics and repeat analysis.
**Figure S3**	Maximum-likelihood phylogeny of mammalian IFN-α and IFN-ω genes showing the shift in the ratio of IFN-α to IFN-ω copy number in bats compared to other mammals.
**Figure S4**	Comparison of the IFN locus in the *Artibeus jamaicensis* long read assembly with a previous short-read assembly based on Illumina reads and 10X data (GenBank accession: GCF_014825515.1)
**Figure S5**	Maximum likelihood phylogeny of the *PRDM9* orthogroup generated using RAxML under the GTRGAMMA model.
**Figure S6**	Multiple alignment of *TP53* showing a bat-specific deletion (codon 320) in the nuclear localization signal domain.
**Figure S7**	Candidate sites under selection in bats in the *LATS2* gene.

Supplementary figures

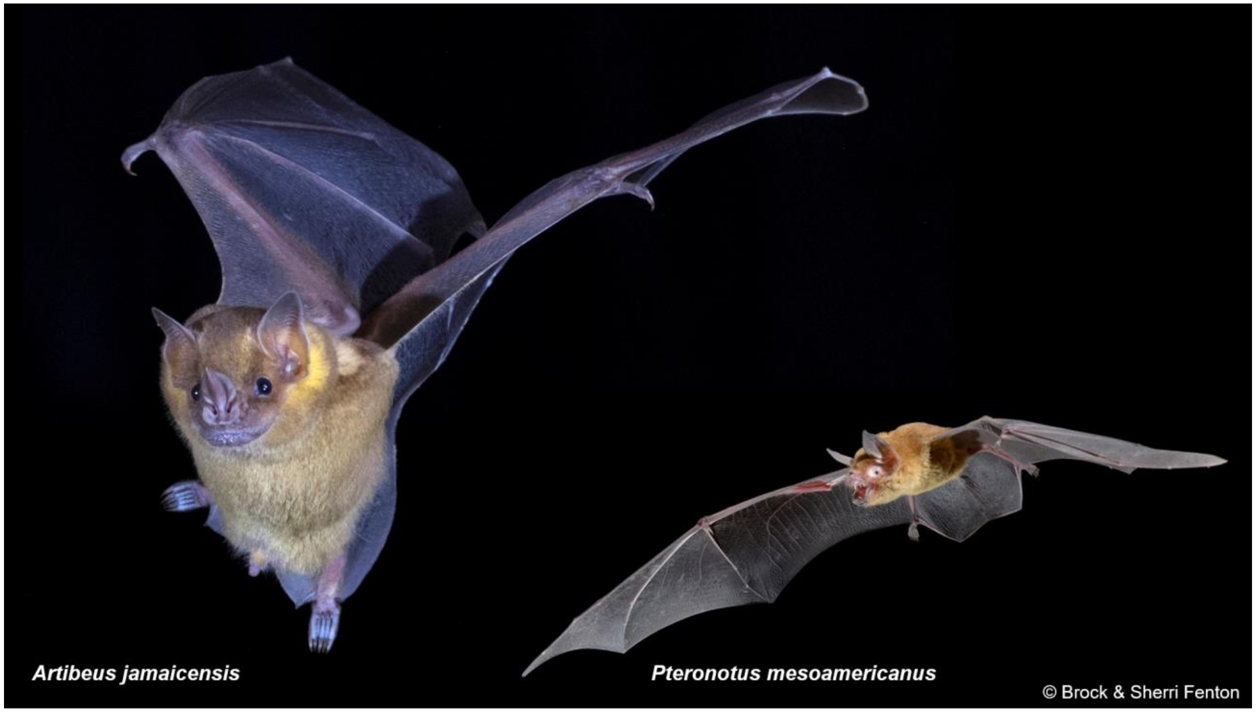

Figure S1. Photographs of the bats *Artibeus jamaicensis* and *Pteronotus mesoamericanus* sequenced in this study. Photographs were provided by Brock & Sherri Fenton.

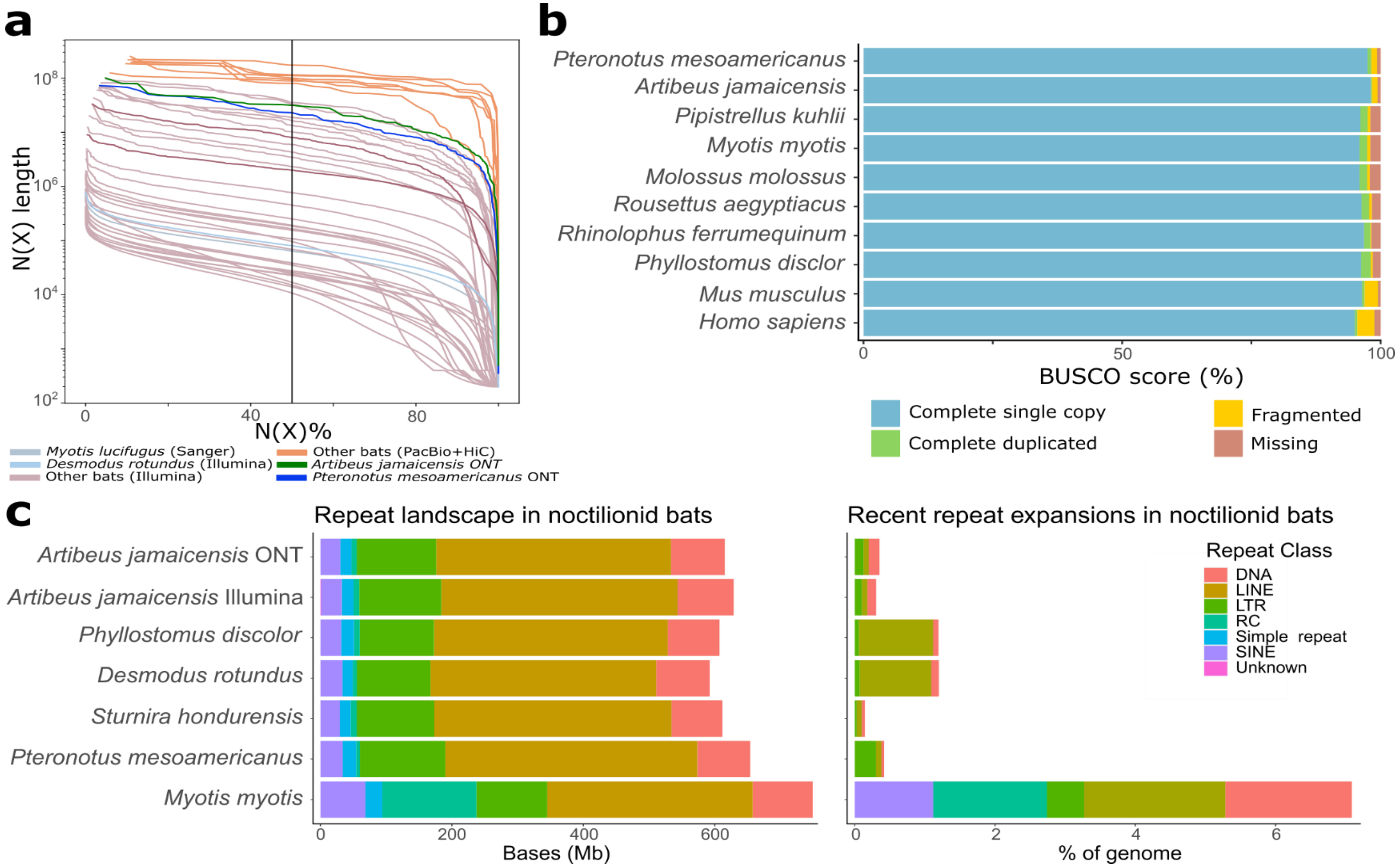

Figure S2. Genome quality statistics and repeat analysis. **a)** Cumulative sum of the length of the ordered contigs versus the cumulative sum of the total proportion of the genome. The vertical black line indicates the N50 metric. The set of ‘Other bats

(PacBio+HiC)’ is provided by the Hiller lab (https://bds.mpi-cbg.de/hillerlab/Bat1KPilotProject/) and ‘Other bats (Illumina)’ are a different set of publicly available bat genomes downloaded from GenBank (see **Table S1** for all GenBank bat assemblies). **b)** Protein-based BUSCO v4 analysis of eight bats as well as human and mouse. The mammalian BUSCO set (odb9) was used. Proteins for bat species not sequenced in this study are based on annotations downloaded from https://bds.mpi-cbg.de/hillerlab/Bat1KPilotProject/. **c)** RepeatMasker analysis of repeat classes in noctilionoid genomes. Noctilionoids exhibit a homogeneous landscape of repeat classes that almost completely lacks the rolling circle (RC) repeats prevalent in *M*. *myotis*. Long interspersed nuclear elements (LINEs) make up the largest repeat class in all bats. Recent repeat expansion make up less than 2% of the genome in all noctilionoids. A list of the recently expanded repeats is provided in **Table S3**.

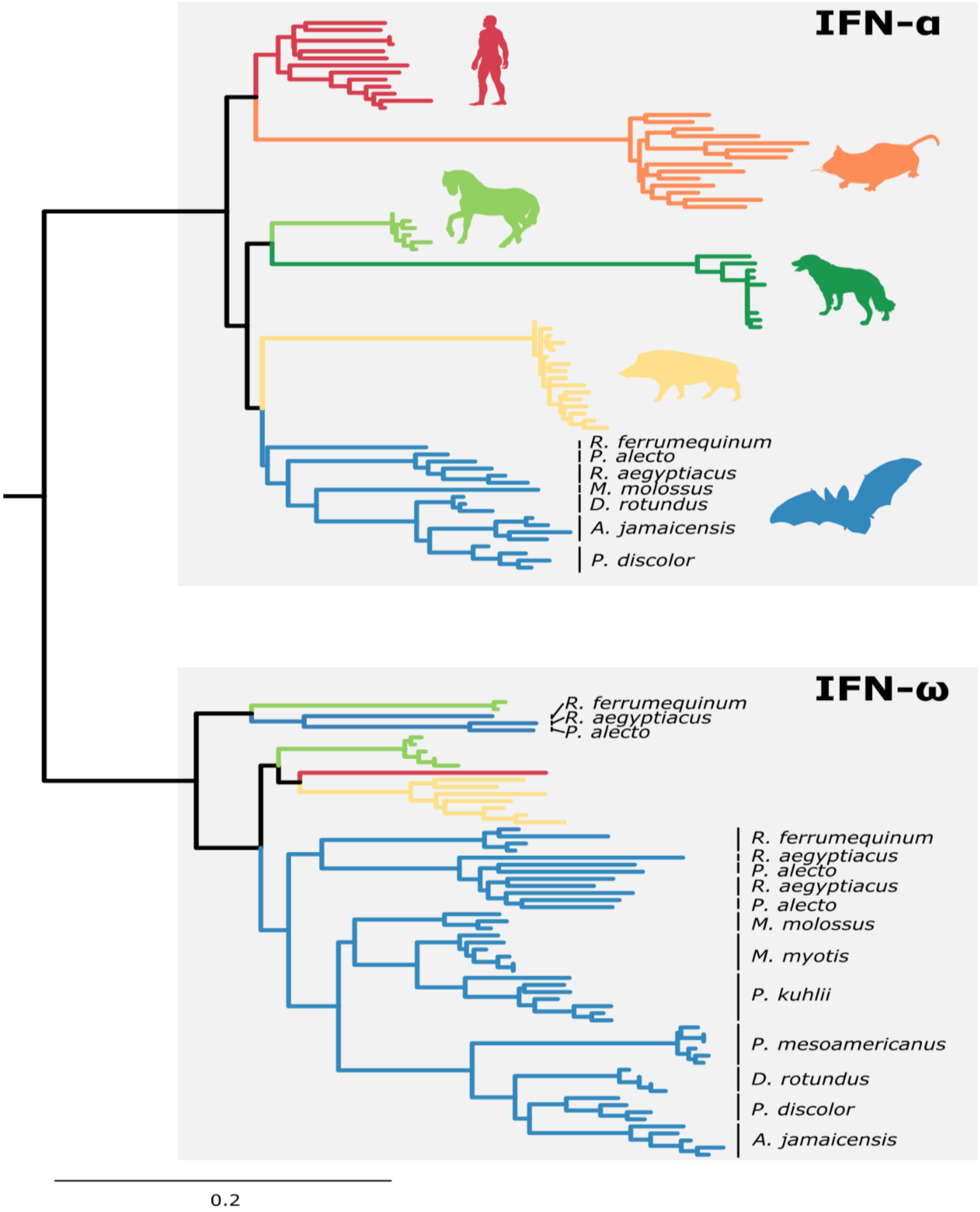

Figure S3. Maximum-likelihood phylogeny of mammalian IFN-α and IFN-ω genes showing the shift in the ratio of IFN-α to IFN-ω copy number in bats (shown in blue) compared to other mammals. The phylogeny was inferred with RAxML under a GTRGAMMA model using alignments partitioned by codon sites. IFN-ω genes are not present in the dog or mouse. Although the topology mostly reflects the expected phylogenetic relationships between species, the tree is intended to show copy number variation in IFN-α and IFN-ω, and it should be noted that internal branches within each ortholog cluster are not robustly supported. The sequence and topology are provided in **Data S3**.

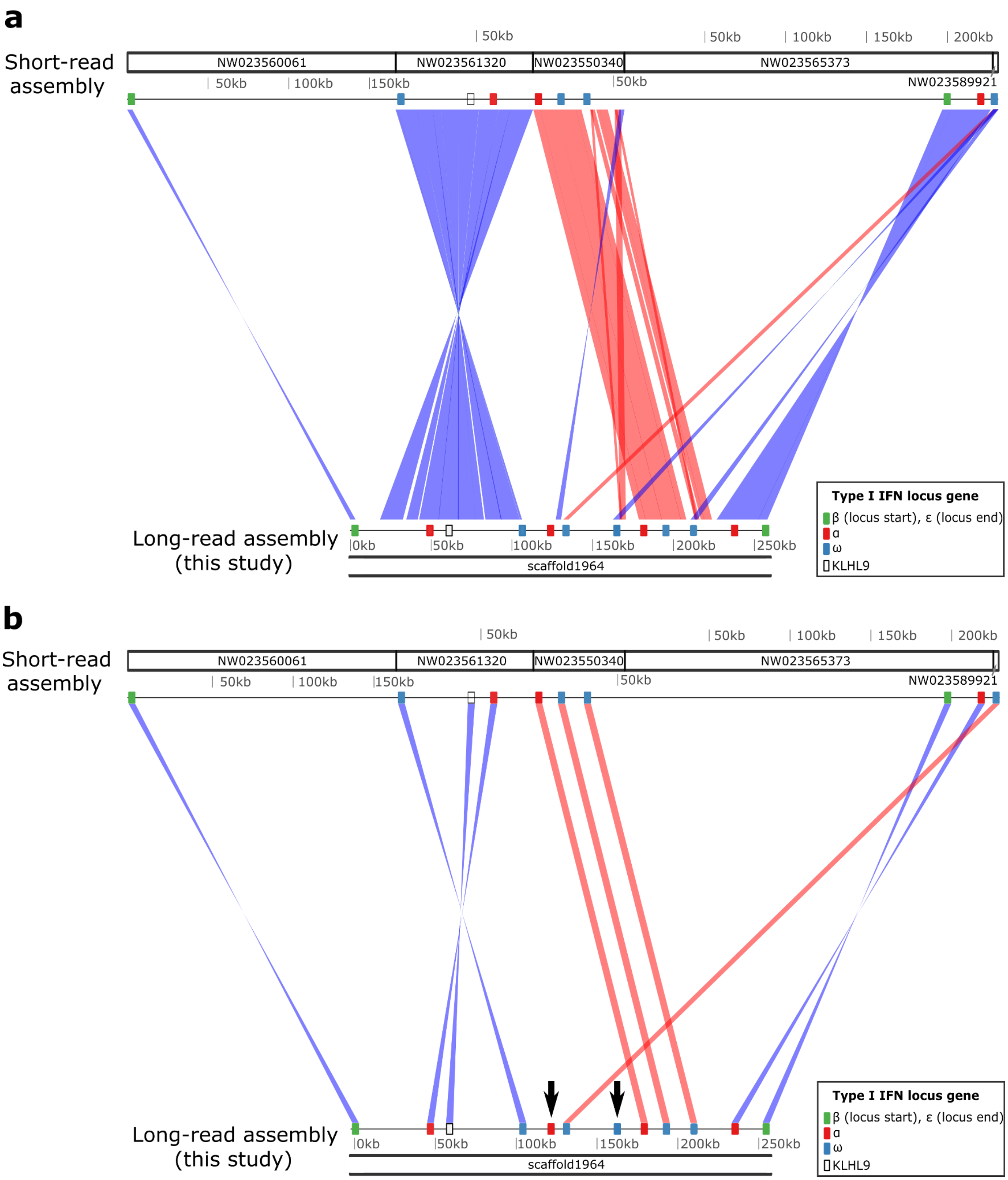

Figure S4. Comparison of the IFN locus in the *Artibeus jamaicensis* long read assembly with a previous short-read assembly based on Illumina reads and 10X data (GenBank accession: GCF_014825515.1). The IFN locus in the short-read assembly is fragmented into 5 scaffolds (NW023560061, NW023561320, NW023550340, NW023565373 and NW023589921) that were concatenated for the comparison. The 2,191bp scaffold NW_023543464.1 was also found to have annotated IFN genes, however this scaffold was excluded as a contaminated scaffold

based on the evidence that the best non-self RefSeq blastp hits for the two proteins encoded on the scaffold were human (accession IDs NP_066401.2 and AAH74965.1). In the long-read assembly, the IFN locus is assembled within the ~73Mb scaffold1964, a region of which is shown. a) Synteny plot between the assemblies based on blastn alignments with >95% identity and an alignment length >1kb. Alignments on the same strand are shown in red and those on the opposite strand are shown in blue. b) Best blastp alignments between proteins encoded on the short-assembly and the long-read assembly. Best alignments were selected based on bit score. Black arrows indicated genes likely missing from the short-read assembly.

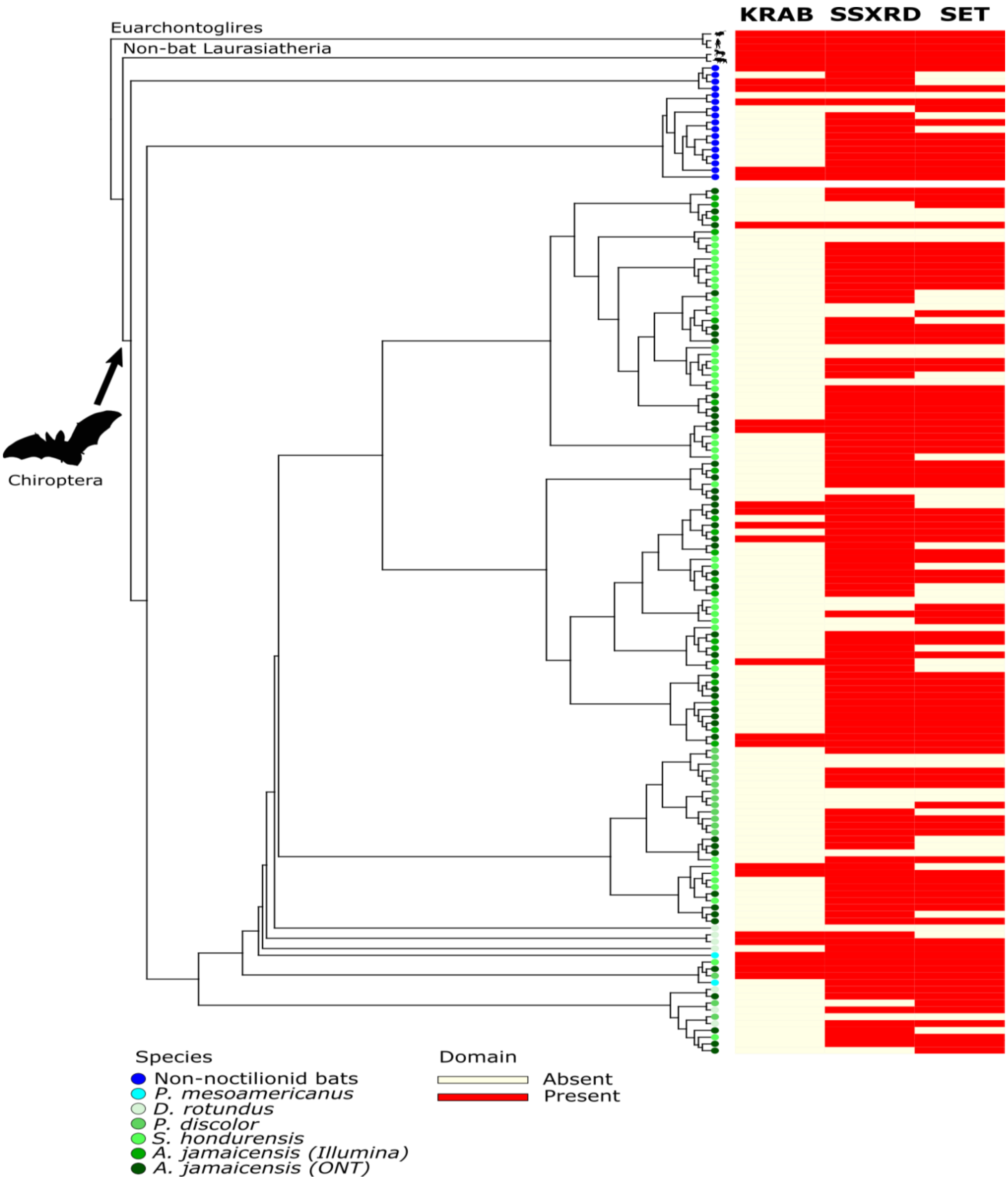

Figure S5. Maximum likelihood phylogeny of the *PRDM9* orthogroup generated using RAxML under the GTRGAMMA model. Presence and absence of the key domains KRAB, SSXRD and SET was determined using pfamscan (https://www.ebi.ac.uk/Tools/pfa/pfamscan/) with default e-value thresholds. The *PRDM9* orthologs underwent an expansion in phyllostomid bats (shown in shades of green). The sequence and topology are provided in **Data S3**.

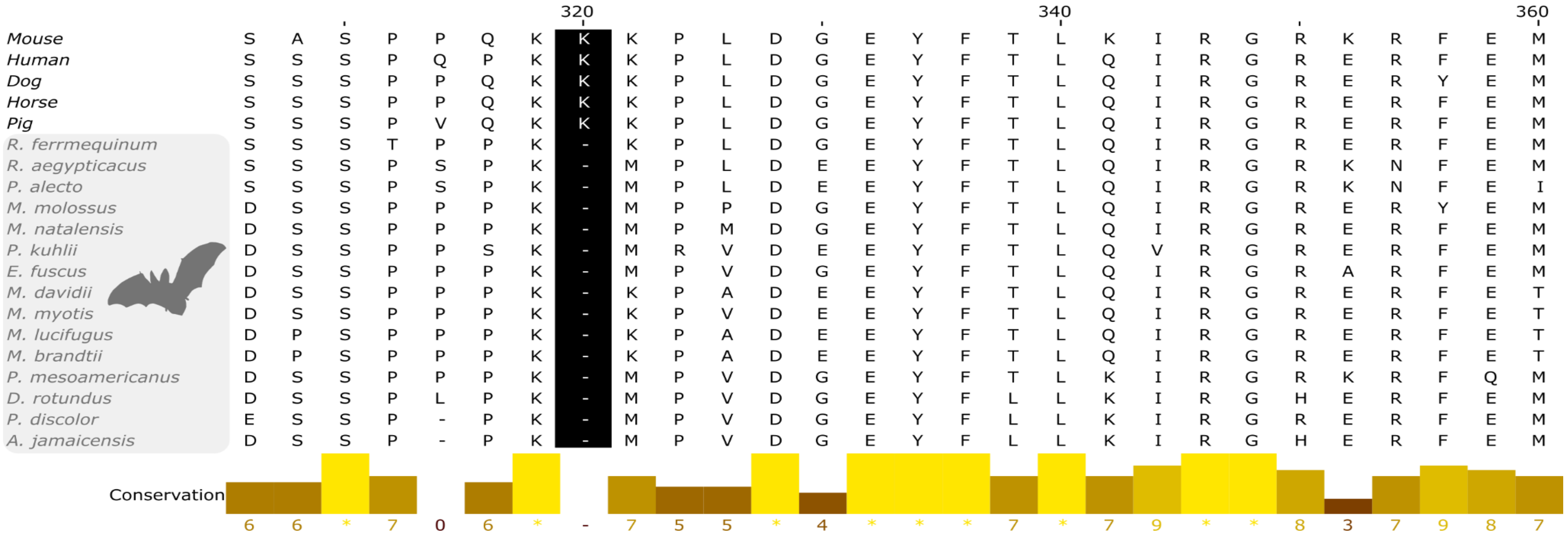

Figure S6. **Multiple alignment of *TP53* showing a bat-specific deletion (codon 320) in the nuclear localization signal domain.** Amino acid positions are based on the human protein (NP_000537.3). Image was exported from JalView 2.11.1.4.

LATS2

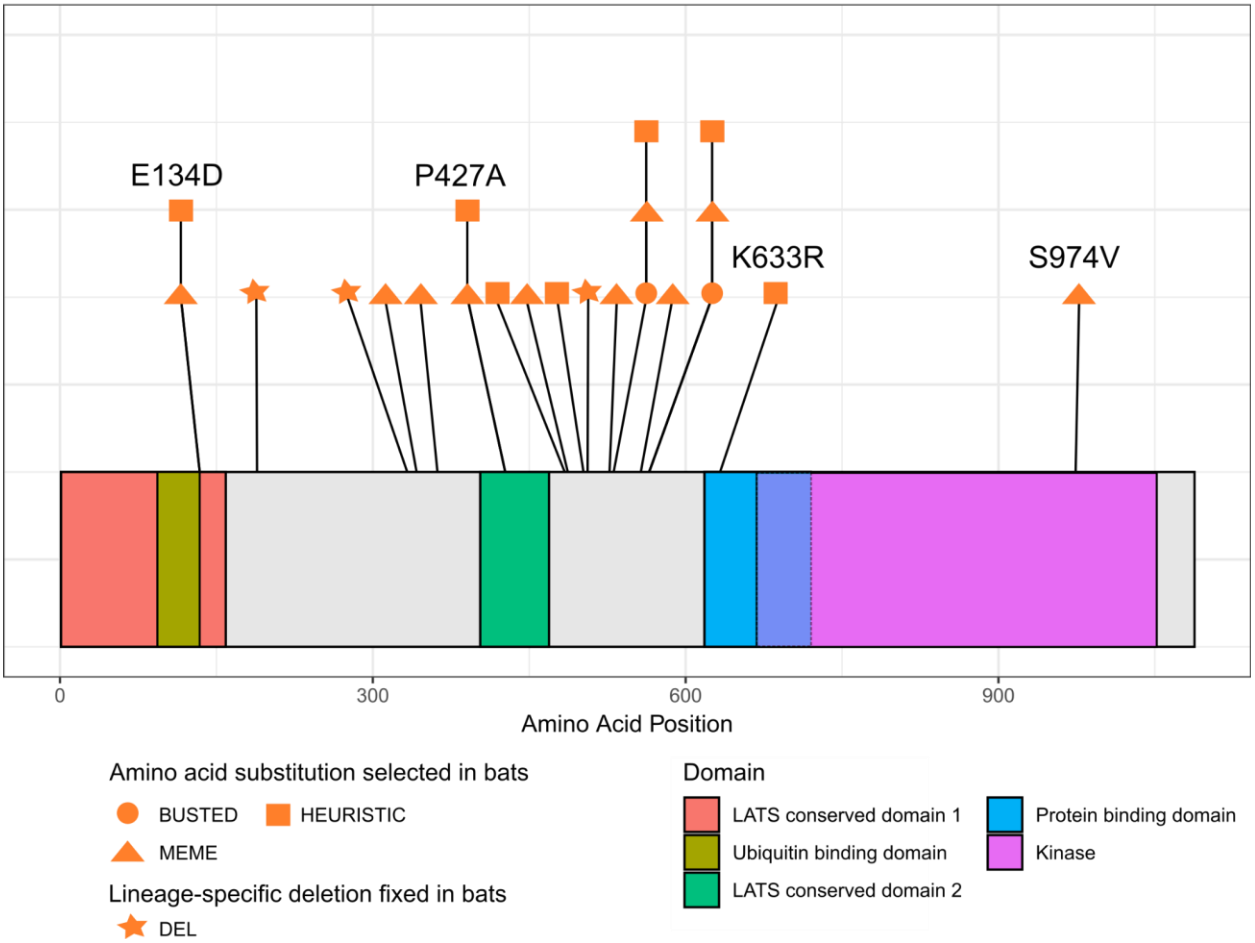

Figure S7. **Candidate sites under selection in bats in the *LATS2* gene.** Substitutions with evidence of selection were identified using BUSTED and MEME as well as a simple heuristic (complete bat-specific fixation of a substitution that is not fixed in the outgroup mammals). Bat-specific deletions (DEL) were observed to begin in codons 189, 333, and 506 (coordinates based on human protein XP_016876030.1). Domain coordinates are based on UniProt (LCD1:1-159, LCD2:403-469, PBD:618-720, KINASE:668-1052, UBA:98-139).
